# Centenarian lifespans of three freshwater fish species in Arizona reveal the exceptional longevity of the buffalofishes (*Ictiobus*)

**DOI:** 10.1038/s41598-023-44328-8

**Published:** 2023-10-20

**Authors:** Alec R. Lackmann, Stuart A. Black, Ewelina S. Bielak-Lackmann, Jeffrey A. Lackmann

**Affiliations:** 1 Department of Mathematics and Statistics, University of Minnesota Duluth, 140 Solon Campus Center, 1117 University Drive, Duluth, MN 55812 USA; 2 Conservation Angler, Phoenix, AZ USA; 3 Department of Biology, University of Minnesota Duluth, 1035 Kirby Drive, SSB 207, Duluth, MN 55812 USA; 4 Department of Biological Sciences, Dept. 2715, North Dakota State University, PO Box 6050, Fargo, ND 58108 USA

**Keywords:** Ecology, Freshwater ecology, Zoology, Ichthyology

## Abstract

During the 1910s three buffalofish species (Catostomidae: *Ictiobus cyprinellus*, *I. bubalus*, *I. niger*) were reared in ponds along the Mississippi River. Individuals of these buffalofishes were transported to locations across the United States to support or establish commercial fisheries, including Roosevelt Lake, Arizona in 1918. During the 1930s–1960s a commercial fishery existed on Roosevelt Lake, ending by 1970. Scarce information exists on Arizona buffalofishes since. From 2018 to 2023 we studied buffalofishes from nearby Apache Lake (adjacent and downstream of Roosevelt Lake) in collaboration with anglers. Here we show that > 90% of buffalofishes captured from Apache Lake are more than 80 years old and that some of the original buffalofishes from the Arizona stocking in 1918 are likely still alive. Using unique markings on old-age buffalofishes, we demonstrate how individuals are identified and inform dozens of recaptures. We now know all species of USA *Ictiobus* can live more than 100 years, making it the only genus of animal besides marine rockfishes (*Sebastes*) for which three or more species have been shown to live > 100 years. Our citizen-science collaboration has revealed remarkable longevity for freshwater fishes and has fundamentally redefined our understanding of the genus *Ictiobus* itself.

## Introduction

Buffalofishes *Ictiobus* spp. are freshwater fishes native to North America that have a complex history. There are five species of buffalofishes in the family Catostomidae^1,2^, and three species are endemic to the Mississippi or Hudson Bay drainages: bigmouth buffalo *I. cyprinellus*, smallmouth buffalo *I. bubalus*, and black buffalo *I. niger*. They are the largest members of the Catostomidae^3^, a group known for its North American diversity and a family for which 55% of species are classified as imperiled^4^. Despite the imperiled nature of catostomids, smallmouth buffalo, bigmouth buffalo, and black buffalo make up a group that have held commercial value^5–7^. Indeed, the first documented buffalofish rearing attempt occurred in the 1880s for commercial purposes^8^. After many failed attempts, the U.S. Fish Commission succeeded and began officially rearing buffalofishes in hatcheries and rearing ponds in the 1910s to supplement the severely declining commercial fishery of buffalofishes^9–15^. Despite these historic efforts for the commercial fishery and rising buffalofish sport fisheries of the twenty-first century^16,17^, buffalofishes are virtually unmanaged (both commercially and recreationally) across most of their USA range today^16,18–21^.

Buffalofishes were introduced to Arizona in 1918 by the Bureau of Fisheries to supplement declining food stocks. Legislators desired to establish a commercial buffalofishery in this region, as new water management practices were taking place^11,22^. Roosevelt Dam, an impoundment along the Salt River in central Arizona, was constructed from 1905 to 1911, which eventually formed Roosevelt Lake^23^. In 1918, an estimated total of 420 buffalofish fingerlings, yearlings, and adults^11^ arrived by rail to Globe, Arizona, likely from the Fairport Biological Station in Iowa^24–26^, and were stocked in Roosevelt Lake^11^. From 1923 to 1930 the lower three reservoirs along the Salt River formed as construction of more dams took place, including Apache (1924–1927), Canyon (1923–1925), and Saguaro (1928–1930) lakes^23^. By the 1930s, buffalofishes were documented in all four reservoirs, but were never documented as an established part of the aquatic community elsewhere along the Salt River or its tributaries^26–28^. For 30 years Roosevelt Lake supported a commercial fishery, which also included common carp *Cyprinus carpio*, while Apache Lake remained virtually unfished^25,28–30^. This was (is) due to its difficult-to-access location along the Apache Trail, via switch-back gravel roads prone to wash out, its steep banks, and fjord-like bathymetry unsuitable for commercial fishing^25,29^. Indeed, there is a paucity of commercial fishing data from Apache Lake, and it is described that the majority of the lake was “untouched”^29^. This differed markedly from Roosevelt Lake, which lies over two flood plains^25,29^.

Little is known about Arizona buffalofishes since the 1970s. New sport fisheries have emerged including catch-and-kill bowfishing, which has raised substantial conservation concern nationwide^16–21,31–33^, but conservation angling of buffalofishes (this study) has not previously been reported. Such activities, in practice, imply new game fish status to these species because they are recreationally pursued. However, fisheries management has not adapted^20,31,34,35^. Buffalofishes were long considered to have low catchability for recreational anglers^1–3^, and because the pejorative “rough fish” label has persisted nationally, it has instilled inaccurate public perceptions of many such fishes^20,21,31,36–38^. As of 2023, no take limits exist on buffalofishes across most of their USA range, including Arizona. Standard funding channels are also shifted away from species that have been traditionally considered nongame, and as a result there is little management of these fishes even as new sport fisheries have emerged^16,19,20,31,34,35,39^.

Recent studies on buffalofishes have uncovered revelational age-validated life history characteristics from careful examination of the otolith (earstone). First, bigmouth buffalo at 112 years of age was discovered and age-validated to be the longest-lived freshwater teleost, a group of more than 12,000 species^16^. A few years later, individuals as old as 127 years with hatch years in the 1890s were discovered^40^, which nearly quintupled their known maximum age prior to 2019 (26 years)^41^. Bigmouth buffalo are one of the longest-lived vertebrates^40^ and are a periodic strategist^42^. The bigmouth buffalo is also capable of migrating long distances^43^, and they have slow growth, delayed maturity, iteroparity, and can exhibit multidecadal gaps in successful recruitment and skip-spawning across years^16,19,40^. Bigmouth buffalo also show negligible senescence and physiological improvements at 100 years old^44^ and accrue external black or orange spots with advanced age^16^. The first black buffalo aged via the otolith was 56 years old^16^, which was more than two times older than previously reported for maximum age (24 years)^1^. Following these results, an Oklahoma state record (for size: 101.5 cm total length and 30.10 kg) smallmouth buffalo *I. bubalus* was aged via the otolith and found to be 62 years old^45^, more than three times older than their maximum age reported prior to 2019 (18 years)^46^. Otoliths were subsequently age validated for smallmouth buffalo^33^. Despite these advances in knowledge, rising buffalofish sport fisheries, and the crucial importance of accurate age information for sustainable fisheries^47^, the age demographics of Arizona buffalofishes are unknown.

In this study we investigate age structure, external pigmentation spots, rod-and-line catchability, recapture dynamics, and population demographics of buffalofishes from Apache Lake, Arizona. Using this information we analyze recruitment over time, determine whether presence or abundance of spots differs across species, and test if the number of orange spots and black spots are correlated within individuals. We also compile buffalofish angling capture events over the past six years from Apache Lake, report on the number of recaptures based on unique markings on individual fish, and track size fluctuations and movement of individuals.

## Methods

### Study site

**Figure 1:**
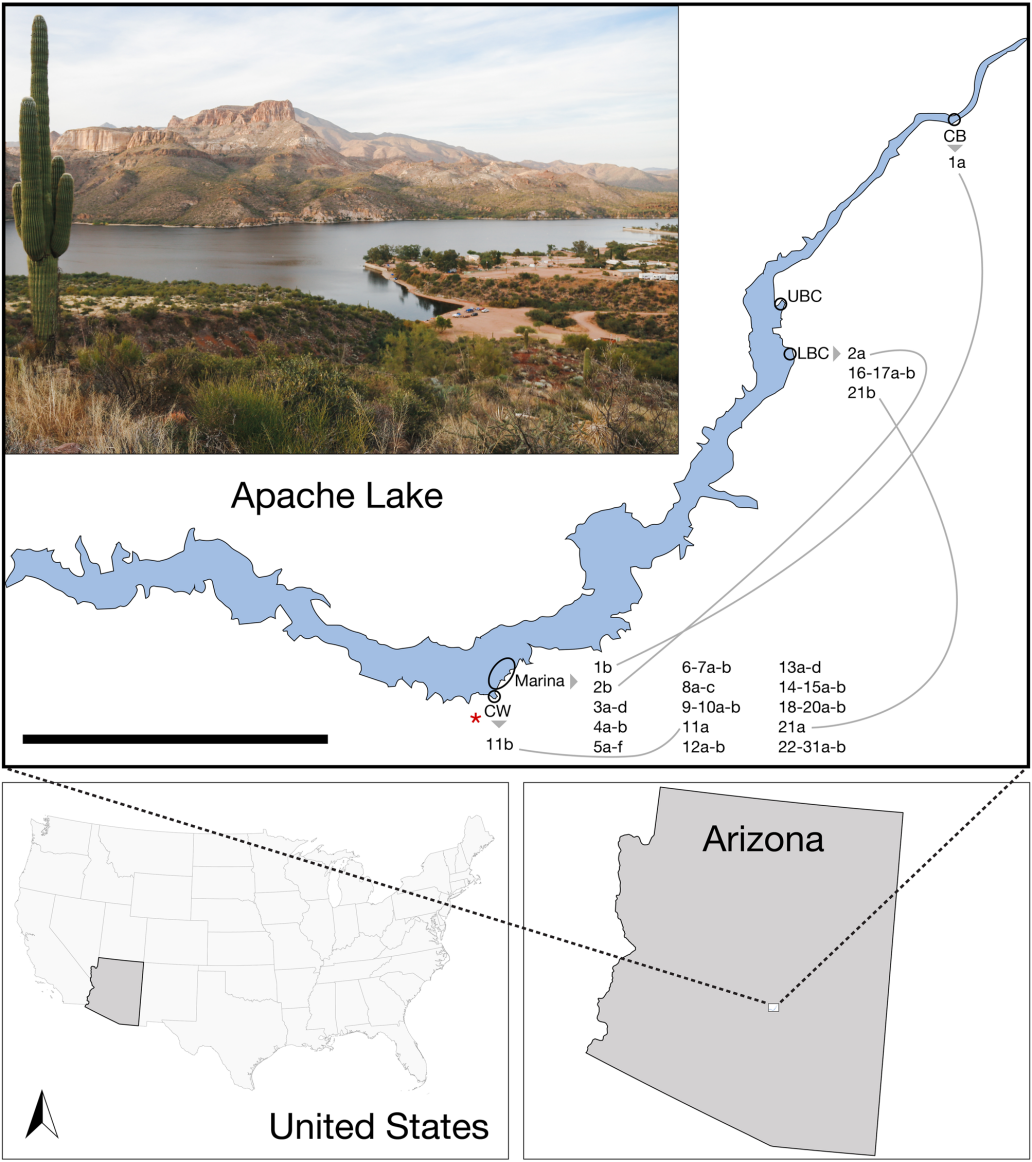
Map of Apache Lake, Arizona, USA showing recreational shore-fishing locations of Crabtree Wash (CW), Marina, Lower Burnt Corral (LBC), Upper Burnt Corral (UBC), and Chunk Beach (CB), as well as the locations where recaptures occurred throughout the study (1a–31b)—see Table 4 (numbers 1–31 represent individual fish, and letters are capture occasions). Picture inset (upper left) is a view of Apache Lake overlooking the Marina from the red asterisk labeled on the map. Buildings of the Marina are visible in the middle-right of the image. Scale bar = 5 km. We retrieved map information for this figure using Google Maps (retrieved April 2023) and organized its layout using Adobe Inc. software including Adobe Illustrator (Creative Cloud version; https://www.adobe.com). The photograph in the upper left was taken by the authors.

Apache Lake (33°34′45.0′′ N 111°15′58.3′′ W) is located approximately 100 km east of Phoenix, Arizona (Fig. 1), and is a narrow, 27 km long, fjord-like reservoir along the Salt River, Arizona. Apache Lake lies downstream of Roosevelt Lake, and upstream of Canyon and Saguaro lakes^24,48^. These four reservoirs make up the Salt River chain of lakes, which is part of the Salt River Project. Apache Lake has a surface elevation of 580 m, and surface water temperature ranges from approximately 8–31 C throughout the year (49). It has a maximum depth of ~ 76 m, a mean depth of ~ 29 m, a maximum length of 28.8 km, and a surface area of ~ 11 km^2^^50^. The biotic community is generally restricted to the upper 10–15 m of the lake^50^, summer stratification is pronounced^24,50^, and, like many reservoirs in Arizona it is almost completely composed of introduced species^24,51^.

### Buffalofish documentation and donation from recreational anglers

From July 2018 to July 2023, recreational anglers documented their buffalofish capture events from Apache Lake using catch-photo-release (CPR) conservation angling standards. In this method of buffalofish angling (Supplementary Fig. 1), anglers use a small (~ 1–2 cm long), micro-barbed hook with a hair-rig for bait presentation. The hook and bait, often a piece of fake corn, are critically balanced for neutral buoyancy amidst pack bait. Anglers deploy pack bait by molding it around their weight before they cast. Pack bait for buffalofishes usually consists of an oat and breadcrumb base to which frozen cladocerans, chironomids, various grubs, corn, and nuts are added, with food oils to round out the mix. As the pack bait settles in the area an angler casts, it creates a food plume. Anglers use alarms set up on rod pods designed to detect subtle bite activity as they wait from shore for hours. Anglers set up and camp along 15 km of the Apache Lake shoreline (Fig. 1) and cast out 10–100 m. Anglers fish in water at depths of anywhere from 1 to 15 m deep, but most often in the range of 5–8 m deep. Anglers catch a variety of species at Apache Lake using this method including gizzard shad *Dorosoma cepedianum*, channel catfish *Ictalurus punctatus*, and common carp. This type of angling was not practiced at Apache Lake until 2017–2018 and has been refined towards buffalofishes in recent years. Buffalofishes were previously considered notoriously difficult to catch on rod and line^1,2,16^, but anglers at Apache Lake have pioneered a consistent method. Overall, this fishing method subtly exploits the invertebrate feeding behavior of buffalofishes that occurs across their lifespan.

For 64.8% of the CPR catch (129 of 199 captures), mass (± 0.005 kg, ± 0.01 kg, or ± 0.03 kg) of the fish was measured by the angler. This was done by weighing the fish in a retention sling (retention sling was tared while empty before placing fish in the sling for weighing). For these angler-weighed fish, we were either present to participate in the weighing, or video footage of the entire taring and weighing procedure was uploaded to social media as part of registering the angler catch. In addition to these CPR captures (*n* = 199), an additional 23 individuals were donated to our research team by the angler instead of being released. Namely, on 17–21 November 2021 recreational anglers donated all buffalofishes captured (*n* = 18 bigmouth buffalo; *n* = 3 smallmouth buffalo) to our research team during an organized buffalofish angling expedition at Apache Lake. This resulted in catch per unit effort (CPUE; defined here as buffalofish per angler per day) = 0.26. Then, during another expedition at Apache Lake from 24 to 30 October 2022, anglers donated black buffalo (*n* = 2) to our research team for further analysis (an additional 28 buffalofishes were released and part of the CPR catch outlined above; *n* = 23 bigmouth buffalo; *n* = 3 smallmouth buffalo; *n* = 2 black buffalo). For this expedition, CPUE = 0.53. More buffalofishes could have been donated for age analysis at the 2022 event to increase sample size, but a precautionary approach was taken. Overall, there was a total of 222 buffalofish capture events across the years of study (2018–2023) with an approximate CPUE of 0.44.

### Body dissections

For the 23 donated fish, we photographed individuals and measured their size, noted and photographed the presence or absence of black or orange spots^16^, dissected gonadal tissue to determine sex, and extracted otoliths for later use in determining age. All animals were treated in accordance with the animal protocol 2305-41079A approved by the University of Minnesota Duluth Institutional Animal Care and Use Committee, and all procedures were carried out in accordance with all relevant guidelines and regulations. This study complies with the ARRIVE (Animal Research: Reporting of In Vivo Experiments) guidelines^52^. We quantified size by wet mass (± 0.005 kg) and total length (± 1 mm) immediately after the fish was landed or was given to our research team (from angler retention slings). After measurement and photographs, fish were euthanized by overanesthetizing with tricaine methanesulfonate. We quantified the number of black or orange spots on each donated buffalofish and measured the largest of each type of spot on each fish using images of each fish on a ruled fishboard. We used ImageJ analysis software^53,54^ to digitally measure the surface area of the largest of each type of spot to the nearest 0.01 cm^2^. We also measured gonadal tissue mass (± 0.01 kg) for all individuals and calculated the gonadosomatic index (GSI = gonad mass/ total body mass). After dissection and extraction we placed otoliths immediately in microvials pre-filled with distilled water. We also photographed, measured size, and noted and photographed orange and black spots for the buffalofishes that were released (*n* = 28) at the October 2022 angling expedition (e.g., Supplementary Fig. 1), except 1 individual that was captured and photographed, and then released before weighing.

### Otolith analysis

In the lab we processed extracted otoliths to obtain photographs of their whole structure. We removed residual cranial tissue, and other non-otolith material under a dissecting microscope. We photographed whole otoliths in water under a dissecting microscope at 50X, using transmitted light in light-field mode. We then air-dried otoliths and calculated lapillus otolith mass using a microbalance (± 0.1 mg) following an established protocol for obtaining buffalofish otolith mass^16,19^. Inspection and photography under a dissecting microscope helped determine the core and primary growth axis of each otolith prior to sectioning.

We then thin sectioned otoliths for age analysis. We embedded otoliths in Buehler epoxy, and then sectioned them using twin diamond-embedded blades on a Buehler IsoMet™ 1000 low-speed saw to produce 300–400 µm sections^16,19,40^. We sectioned otoliths through the core and along the primary growth axis. We mounted sections on a glass slide, immersed them in mineral oil, and photographed them at 75X on a compound microscope. We produced a total of 26 thin sections (i.e., 26 otoliths were sectioned) across all fish (*n* = 23). That is, for 20 individuals one otolith was sectioned, whereas for 3 individuals two otoliths were sectioned.

**Figure 2:**
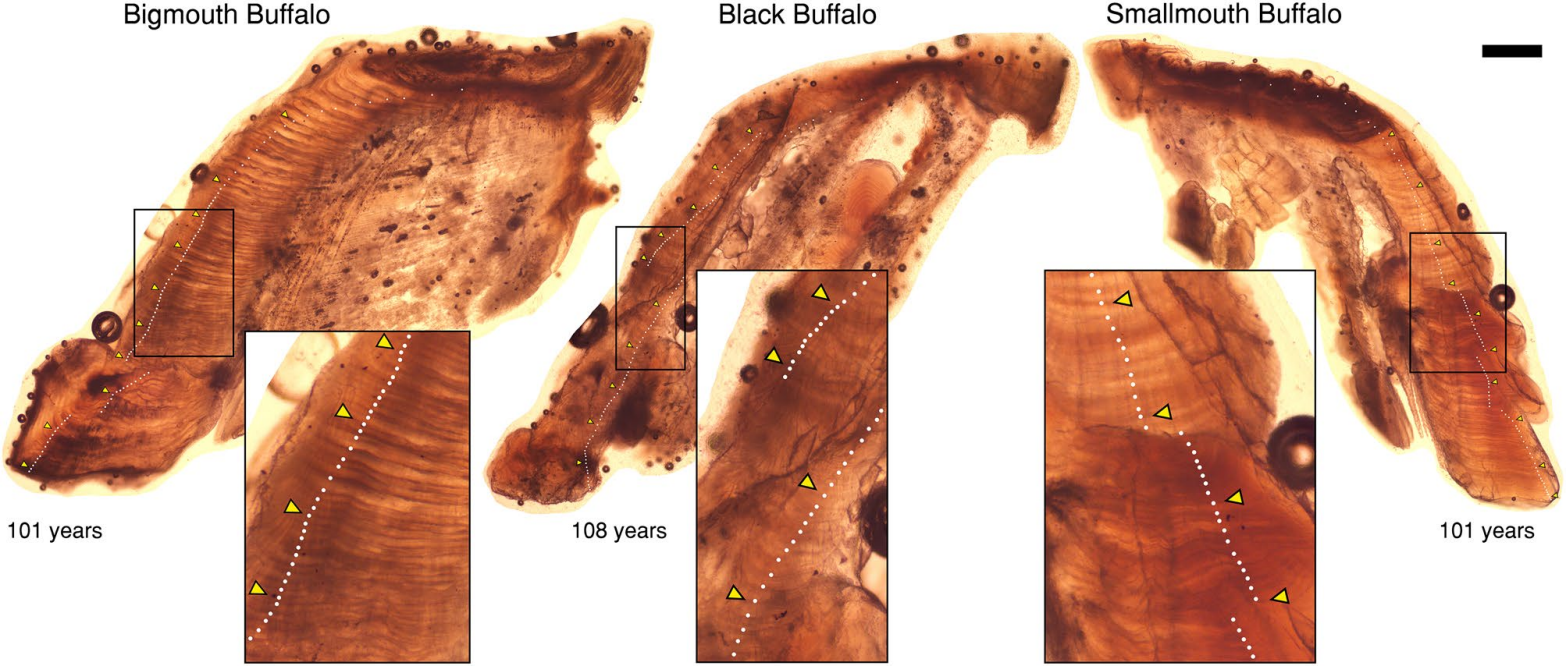
Thin-sectioned asteriscus otoliths (with insets) of Arizona buffalofishes. Examples are of an estimated 101-year-old male bigmouth buffalo *Ictiobus cyprinellus*, a 108-year-old male black buffalo *I. niger*, and a 101-year-old male smallmouth buffalo *I. bubalus* from Apache Lake, Arizona. Fish were collected in 2021 (*I. cyprinellus* and *I. bubalus*) and 2022 (*I. niger*). White dots note annuli; triangles mark decades. Scale bar = 600 μm (does not apply to insets).

We analyzed images of thin sections to determine individual age. Annuli were digitally marked on images by independent readers following an established protocol^16,19,40^. Age readers have experience dissecting, processing, and age-scoring thousands of buffalofish otoliths. Otoliths have been age validated for bigmouth buffalo in multiple ways following recommendations for thorough age validation^17^. Long-lived individuals were age-validated within and across individuals using bomb radiocarbon dating^16^, and early growth annuli were validated using edge analysis^19^. Otoliths are the most accurate structure for age analysis in fishes^47,55,56^. Furthermore, Long et al.^33^ recently age-validated otoliths of smallmouth buffalo, and the validated age-reading protocol that we developed for bigmouth buffalo^16,19,40^ was readily applied to the other buffalofish species because of the otolith’s clear homologous structure (Fig. 2). We assigned year classes to fish based on collection date and ages derived from the total annuli marked on the thin-sectioned otolith images. For each image, we did not count the edge of the otolith thin section as an annulus because these were fall collected fish and buffalofishes are known to spawn once during spring^16,19,40,57,58^.

### All-encompassing Apache buffalofish photo database

We organized and compiled all angler-photographed buffalofishes caught and released at Apache Lake from July 2018 to July 2023 in addition to the buffalofishes that we photographed and measured directly (previous paragraphs). We organized the database chronologically, noted species, location caught (within Apache Lake), the size (if measured), and by whom. We linked all fish capture events (*n* = 222) to their corresponding photos, except in rare cases (2.3%) when photos were not available. For these rare cases we identified catch date, location, and species by either witnessing their catch, the social media post of the catch when it occurred, or we spoke directly to the angler. Once compiled, we systematically reviewed all fish photos for identification of unique pigmentation markings and features that could reveal recaptured individuals^16^. A recaptured individual was confirmed when at least three diagnostic features (e.g., different orange or black spots, lateral line sinuosity, scalation pattern, fin deformities, etc.) were present as an exact match across capture events, and multiple expert observers agreed that it was a recapture. Using this protocol, the possibility of false positives was greatly reduced and was presumed zero because it selectively removed pictures for which diagnostic features were not evident due to poor image quality or lack of photos^59–64^. Indeed, there was no case for which a recaptured individual was identified by one observer but was not in agreement by the other observers. However, the possibility of some false negatives is presumed because some individuals were only photographed on one side. Thus, our estimate of the total number of recaptures is likely an underestimate.

We repeated the process of systematically examining the photo database approximately 80 total times (reviews of all the images each time). For the first 40 times recaptures were consistently identified (as frequent as at least 1 recapture discovered every review, to as infrequent as 1 recapture discovered every fourth review). As recaptures were discovered they were moved into their own recapture subfolder. This recapture subfolder was reviewed in tandem with the non-recapture subfolder. After approximately the 40th time it became very difficult to identify any more recaptured individuals, with only a few more identified after this point. We concluded review of the non-recapture and recapture subfolders after 15 reviews in a row for which no additional recaptures were identified.

We analyzed spot pattern across individuals in the all-encompassing photo database to determine if spot pattern may differ by species for apparently old-age and confirmed old-age individuals. We restricted spot pattern analysis to those individuals for which both sides of the fish had been photographed. We defined apparently old-age individuals as those that had external pigmentation spots related to old age^16^ but were not sacrificed for direct age analysis via the otolith. Confirmed old-age individuals were defined as those that were sacrificed for direct age analysis via the otolith, and were found to be > 40 years old. We noted whether both sides of the fish were photographed or if only one side of the fish was photographed, noted categorically (Yes or No) if the individual had black spots, as well as if (Yes or No) the individual had orange spots, and then we quantified the number of each type of spot for each fish.

### Statistical analysis

We calculated the average coefficient of variation (CV) of age reader scores, and the average CV for each species^65^ for measure of age estimate precision. We analyzed recruitment patterns for bigmouth buffalo using contingency analysis^19,40^. Using sample data, we defined “evidence of recruitment” categorically (Yes/No) for each year 1916–2021, based on whether a 2021 collected bigmouth buffalo was from that respective year class. We used 1916 as the starting point because that is the earliest bigmouth buffalo year class in the sample. We then tested whether evidence of recruitment in a given year is independent of evidence of recruitment the previous year.

We characterized the distribution of buffalofish external black or orange spots (count data on the number of spots per individual) using Poisson and negative binomial, and used Akaike’s information criterion corrected for sample size (ΔAIC_c_)^66,67^ to select the more parsimonious model of the overall distribution. We then compared the distribution of spots across species through a generalized regression of species and the more parsimonious (i.e., Poisson or negative binomial) error distribution. For fully photographed individuals analyzed for spot pattern, we grouped buffalofishes into three groups: (1) those that were aged directly (Aged), (2) those that were caught and released at the October 2022 angling expedition (Oct22R), and (3) those that were caught and released at the remaining outings (Other). We analyzed spot patterns across these groups to test if the number of spots differed across outing type, principally because we photographed the individuals in (1) and (2), but not always for (3)—anglers photographed their catch in group (3). We also used contingency analysis to test whether the presence or absence (Y/N) of orange spots differed significantly by species. We used a t-test of the Pearson product-moment correlation coefficient (*r*) to determine if the number of orange spots and black spots is correlated in buffalofish individuals. We used JMP^®^ Pro Version 16 (SAS Institute, Inc., Cary, NC 1989–2023) software for statistical analyses.

## Results

We estimated the ages of all 23 buffalofishes donated to our research team from annulus counts of the otolith thin sections. Ages ranged from 85 to 105 years old for bigmouth buffalo (*n* = 18), 11–101 years old for smallmouth buffalo (*n* = 3), and 106–108 years old for black buffalo (*n* = 2) (Fig. 2). The overall between-reader aging precision had a coefficient of variation (CV) of 4.0%, with a CV of 3.9% for bigmouth buffalo, 4.4% for smallmouth buffalo, and 2.4% for black buffalo. Size of these aged bigmouth buffalo ranged from 68.4 to 88.5 cm TL and 3.91–12.67 kg in mass, and there were 7 females and 11 males (Table 1). Size of these aged smallmouth buffalo ranged from 75.3 to 86.5 cm TL and 6.97–13.47 kg in mass, and there were 2 females and 1 male (Table 1). Size of these aged black buffalo ranged from 74.8 to 88.9 cm TL and 4.65–11.85 kg in mass, and there was 1 female and 1 male (Table 1).

**Table 1:** Demographic characteristics of aged Apache Lake, Arizona buffalofishes (*n* = 23) collected by anglers in 2021 and 2022.

Species	Capture date	Age	YC	OM a	OM b	Mass	Sex	G	TL	GSI	#B	#O	LB	LO
*I. bubalus*	20-Nov-21	91	1930	106.1	99.8	13.47	F	2.00	86.5	0.148	9	0	0.38	
*I. bubalus*	21-Nov-21	11	2010	32.5	31.5	8.73	F	1.31	79.0	0.150	0	0		
*I. bubalus*	18-Nov-21	101	1920	111.0	108.9	6.97	M	0.51	75.3	0.073	6	1	0.65	0.10
*I. cyprinellus*	21-Nov-21	98	1923	151.7	134.3	9.41	F	1.70	88.5	0.181	23	2	0.91	0.61
*I. cyprinellus*	18-Nov-21	105	1916	121.0	115.9	11.25	F	0.37	88.1	0.033	6	1	6.70	4.70
*I. cyprinellus*	18-Nov-21	98	1923	115.7	108.1	12.67	F	2.00	87.1	0.158	2	0	6.14	
*I. cyprinellus*	20-Nov-21	99	1922	99.4	91.6	8.65	F	1.50	85.9	0.173	7	10	0.28	0.85
*I. cyprinellus*	18-Nov-21	96	1925	111.7	104.1	9.95	F	1.69	83.6	0.170	7	2	2.10	50.04
*I. cyprinellus*	18-Nov-21	86	1935	143.8	142.3	9.16	F	1.39	83.6	0.152	3	1	0.29	0.26
*I. cyprinellus*	17-Nov-21	98	1923	107.3	102.9	9.41	F	1.33	80.4	0.141	5	0	6.26	
*I. cyprinellus*	20-Nov-21	99	1922	115.1	109.5	5.98	M	0.47	81.8	0.079	10	4	3.69	0.17
*I. cyprinellus*	21-Nov-21	99	1922	109.9	103.4	5.95	M	0.32	79.6	0.054	11	6	1.90	1.12
*I. cyprinellus*	20-Nov-21	101	1920	102.7	96.4	6.10	M	0.45	76.0	0.074	14	3	36.53	0.28
*I. cyprinellus*	19-Nov-21	85	1936	114.8	105.2	4.70	M	0.17	75.0	0.036	6	4	0.79	0.91
*I. cyprinellus*	18-Nov-21	104	1917	134.8	123.7	4.96	M	0.12	74.5	0.024	15	10	2.64	70.00
*I. cyprinellus*	20-Nov-21	101	1920	118.3	113.8	6.12	M	0.53	74.1	0.087	20	3	4.03	1.69
*I. cyprinellus*	19-Nov-21	98	1923	113.9	105.0	5.52	M	0.38	72.6	0.069	7	0	0.41	
*I. cyprinellus*	19-Nov-21	98	1923	132.1	126.1	5.41	M	0.14	72.2	0.026	3	1	0.30	0.03
*I. cyprinellus*	20-Nov-21	98	1923	75.4	71.2	3.91	M	0.04	69.9	0.010	5	1	0.26	0.62
*I. cyprinellus*	19-Nov-21	95	1926	97.9	83.1	4.56	M	0.30	68.7	0.066	16	1	3.14	0.47
*I. cyprinellus*	19-Nov-21	97	1924	99.9	96.1	4.73	M	0.44	68.4	0.093	10	2	0.76	1.51
*I. niger*	28-Oct-22	106	1916	140.9	114.1	11.85	F	2.64	88.9	0.223	16	0	13.55	
*I. niger*	25-Oct-22	108	1914	92.0	85.2	4.65	M	0.06	74.8	0.012	3	0	0.10	

Data are sorted by species, then by sex, and then by size (cm) in total length (TL). YC = year class; OM a = largest otolith mass (OM) in mg of the two lapilli; OM b = smallest OM of the two lapilli; Mass and Gonad (G) in kg; GSI = gonadosomatic index expressed as a relative frequency; #B = number of black spots; #O = number of orange spots; LB = largest black spot (cm^2^); LO = largest orange spot (cm^2^).

**Figure 3:**
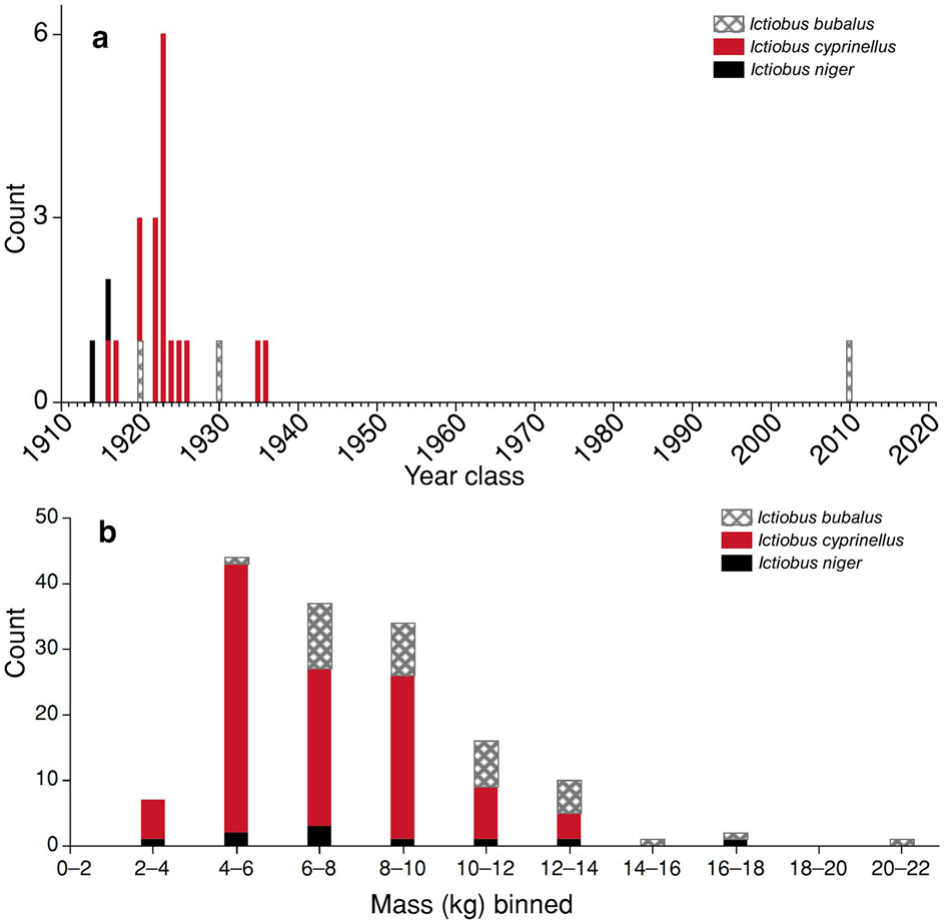
Year class and size distribution of buffalofishes from Apache Lake, Arizona in this study. **a** Year class distribution estimated via ages from thin-sectioned otoliths collected from anglers in 2021 (*I. cyprinellus*: *n* = 18; *I. bubalus*: *n* = 3), and in 2022 (*I. niger*: *n* = 2). For bigmouth buffalo, the species with the largest sample size, year classes (red bars) range from 1916 to 1936, and the 1923 year class was most abundant composing 33% of the sample. **b** Size distribution of buffalofishes (including recaptures) measured for mass across all years of the study (2018–2023) binned in 2 kg intervals (*I. cyprinellus*:* n* = 108; *I. bubalus*:* n* = 34; *I. niger*:* n* = 10). See Tables 1–4 for more details.

Year classes ranged from 1916 to 1936 for bigmouth buffalo, with 78% of the fish coming from 6 year-classes during the 7-year span of 1920–1926 (Fig. 3a). We found no evidence of bigmouth buffalo recruitment in Apache Lake during the past 8.5 decades (Fig. 3a). Evidence of bigmouth buffalo recruitment observed in one year was not independent of evidence of recruitment in the previous year. A likelihood ratio test indicated that the distribution of recruitment between years was not random (χ^2^ = 21.3, df = 1, *n* = 105, *p* < 0.0001, *R*^2^ = 0.35). That is, if evidence of recruitment is not observed, it is 97% likely to not be observed again the following year. Conversely, if evidence of recruitment is observed in a year, then it is 60% likely to be observed the following year. We estimated the three smallmouth buffalo were from year classes 1920, 1930, and 2010, and the two black buffalo were from year classes 1914 and 1916. We also found that the size distribution of all buffalofishes measured for mass (*n* = 152) was unanimously composed of individuals of mature sizes (Fig. 3b). That is, bigmouth buffalo ranged in size from 2.32 to 13.01 kg (median = 6.49 kg; *n* = 108), smallmouth buffalo from 5.93 to 20.38 kg (median = 9.14 kg; *n* = 34), and black buffalo from 2.44 to 17.86 kg (median = 6.35; *n* = 10) (Fig. 3b). There is significant sexual dimorphism of buffalofishes at adult size^16,19,40^ (Table 1).

**Figure 4:**
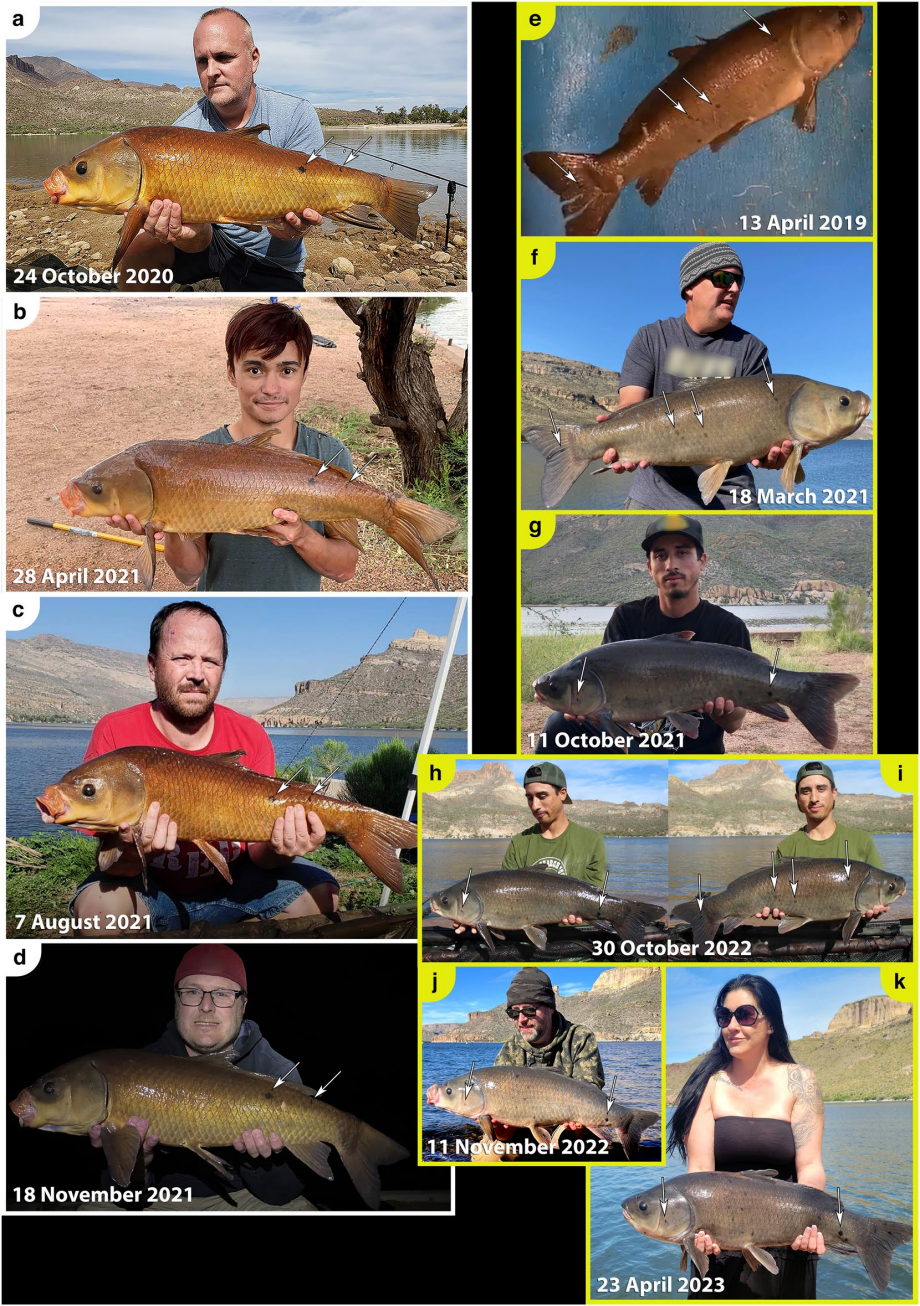
Examples of multi-capture sequences of individual bigmouth buffalo *Ictiobus cyprinellus* from Apache Lake, Arizona across years. (**a**)**–**(**d**) An individual male was caught by four anglers on four dates across a 13-month period. This specimen (RCID#13 in Table 4) ranged from 4.96 to 5.30 kg across captures and was noted for two prominent black markings on its left side (dorsal posterior, inset arrows **a**–**d**), as well numerous other black and orange spots not visible from these full-body images. (**e**)–(**k**) An individual bigmouth buffalo was caught six times by five anglers across a 4-year period. This specimen (RCID#5 in Table 4) ranged from 5.56 to 6.83 kg and was noted for its numerous black spots (see inset arrows in **e**–**k** for examples).

**Figure 5:**
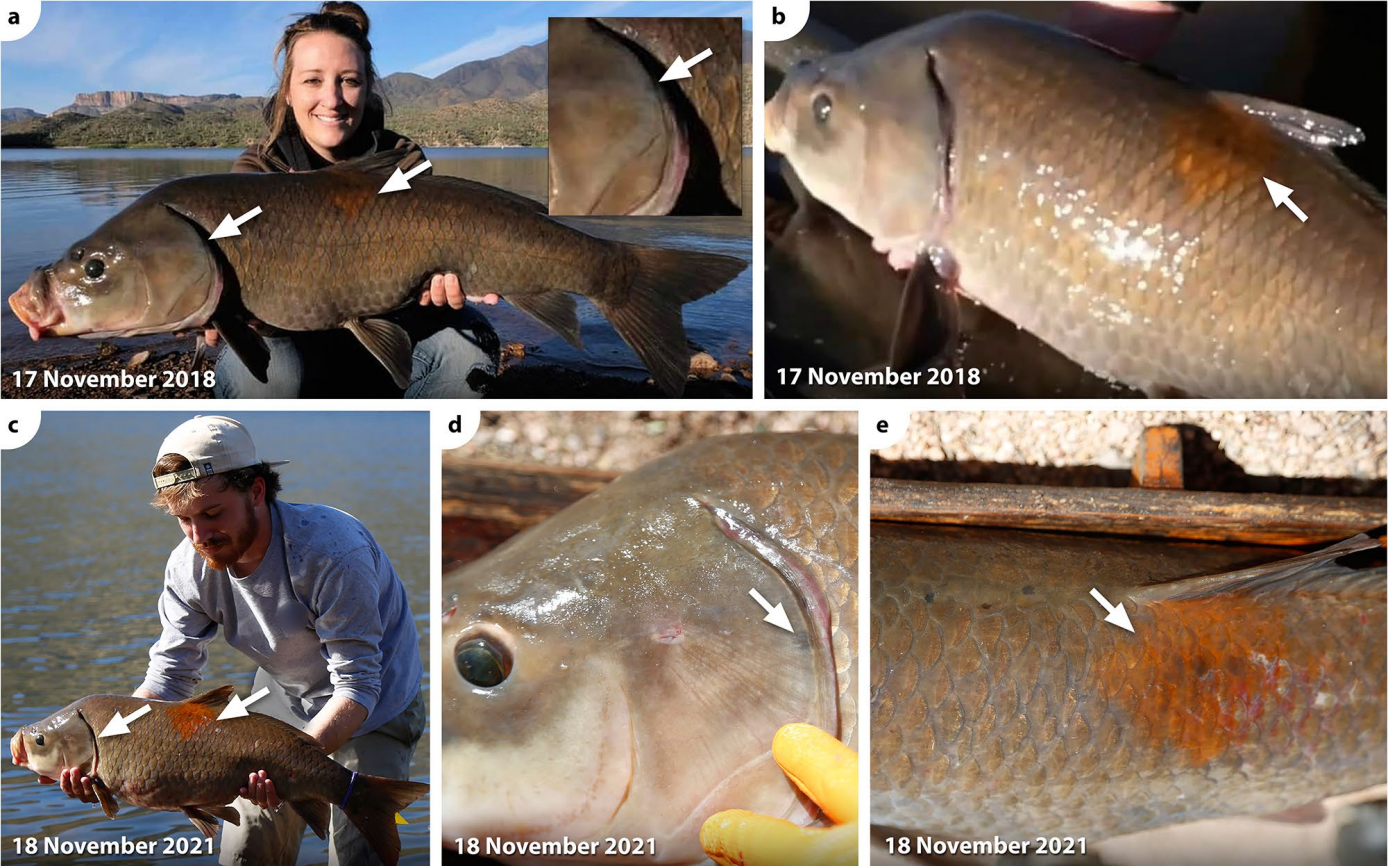
Natural orange and black spots provide unique identifiable markers on an old-age buffalofish individual from Apache Lake, Arizona. This female bigmouth buffalo *Ictiobus cyprinellus* was first caught in 2018 (**a–b**), and then caught a second time in 2021 (**c–e**). This specimen was noted for a large orange spot (~ 50 cm^2^) on its left side (see middle arrow in **a**, arrows in **b**, **c** and **e**), as well as a black spot on the posterior margin of its left operculum (see other arrows in **a**, **c**, and arrow in **d**). This specimen (RCID#2 in Table 4) increased from 7.03 to 9.95 kg and the distance between captures was 7.5 km (see Fig. 1).

**Figure 6:**
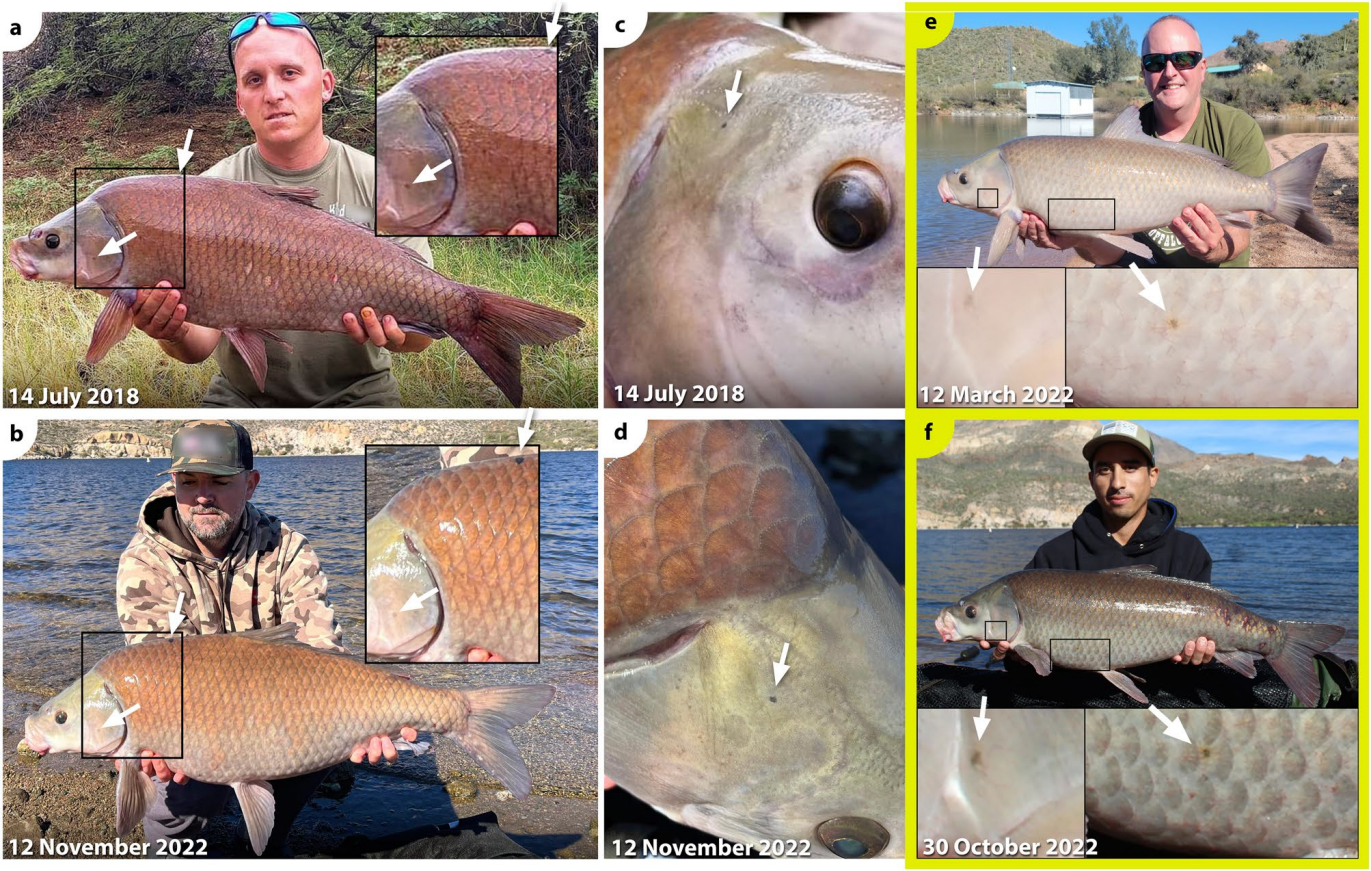
Smallmouth buffalo *I. bubalus* and black buffalo *I. niger* recaptures from Apache Lake, Arizona. An individual smallmouth buffalo (RCID#1 in Table 4) was caught twice in 4.33 years (**a–d**). This specimen was noted for black spots on its left operculum and dorsal anterior (see arrows and insets in **a–b**), as well as a black spot on its head, posterior and dorsal of its right eye (see arrows **c–d**). This specimen (RCID#2 in Table 4) increased from 7.17 to 9.41 kg and the distance between captures was 13.0 km (see Fig. 1). (**e–f**) An individual black buffalo (RCID#25 in Table 4) was caught twice across a 7-month period. This specimen was noted for black spots on its left side (see arrows and insets in **e–f**). This specimen decreased from 6.46 to 6.24 kg (see Table 4) across captures.

*Ictiobus* from Apache Lake exhibit a striking amount of phenotypic variation as evidenced from black or orange spots. All buffalofishes from the November 2021 and October 2022 angling expeditions, except the 11-year-old smallmouth buffalo, had either black or orange spots. All 18 bigmouth buffalo had black spots, ranging in number from 2 to 23 spots depending on the individual (Table 1, Figs. 4, 5). The largest black spot on each of these bigmouth buffalo ranged from 0.26 to 36.50 cm^2^ (Table 1). We also found evidence of orange spots on 15 of 18 of these bigmouth buffalo, ranging in number from 1 to 10 spots (Table 1, Figs. 4, 5). The largest orange spot on each of these bigmouth buffalo ranged from 0.03 to 70.00 cm^2^ (Table 1). The three individuals that lacked orange spots consisted of two females and one male, and they were all estimated to be 98 years old (Table 1). We found that 2 of 3 smallmouth buffalo had evidence of black spots ranging from 6 to 9 spots (Table 1, Fig. 6). The largest black spot on each of these individuals was 0.38 cm^2^ and 0.65 cm^2^ (Table 1). There was one orange spot on the 101-year-old smallmouth buffalo that was 0.10 cm^2^ in size. The 11-year-old smallmouth buffalo showed no evidence of black or orange spots. The black buffalo that were aged had black spots ranging from 3 to 16 spots per individual, but no orange spots. The largest black spot on each of these individuals was 0.10 cm^2^ and 13.55 cm^2^ (Table 1). Black or orange spots were not previously described for black buffalo or smallmouth buffalo, but had been recently described for bigmouth buffalo^16^.

**Figure 7:**
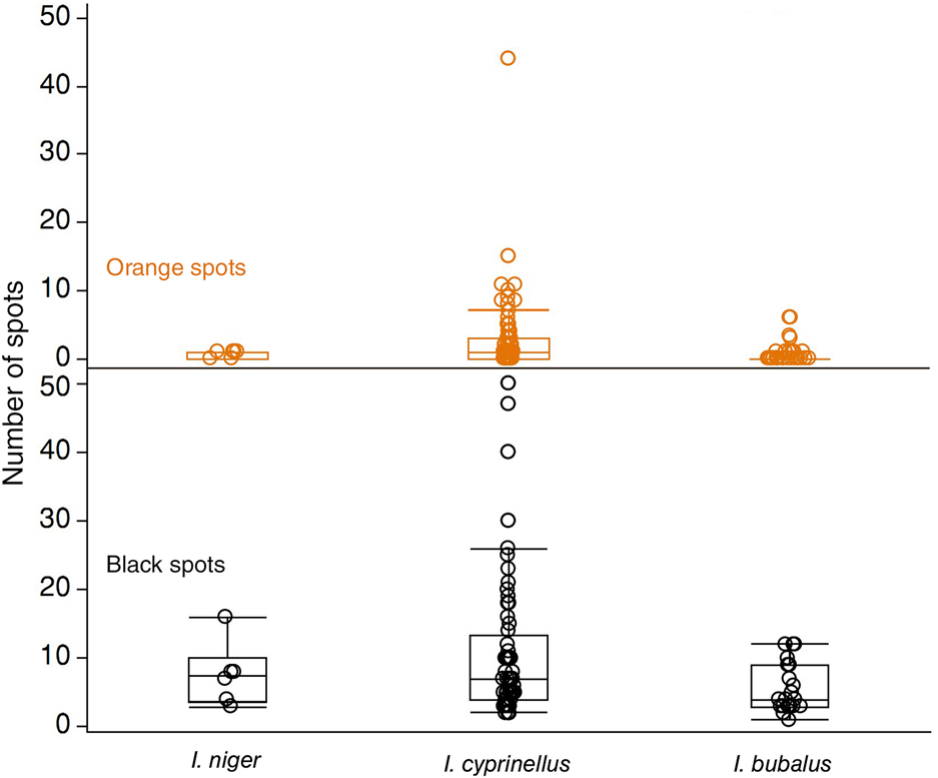
Number of external pigmentation spots by type of spot (with box plots), across fully photographed apparently old-age or confirmed old-age individuals of three buffalofish species: black buffalo *I. niger* (*n* = 6), bigmouth buffalo *I. cyprinellus* (*n* = 68), and smallmouth buffalo *I. bubalus* (*n* = 23) from Apache Lake, Arizona.

Buffalofish spot patterns were consistent across outing type. In addition to the 23 aged buffalofishes, another 75 individuals across 107 capture events (numerous recaptures) were photographed on both sides and the number and type of external pigmentation spots were quantified (Tables 2, 3). These included bigmouth buffalo (*n* = 50), smallmouth buffalo (*n* = 21), and black buffalo (*n* = 4). For these additional 75 fully photographed individuals, every fish had at least one black spot (Tables 2, 3). The number of black spots ranged from 2 to 50 on bigmouth buffalo, 1–12 on smallmouth buffalo, and 4–8 on black buffalo; whereas orange spots were not as numerous but were found on all species (Tables 2, 3). The number of orange spots ranged from 0 to 44 on bigmouth buffalo, 0–6 on smallmouth buffalo, and 0 to 1 on black buffalo (Tables 2, 3). Overall, there were 98 unique buffalofishes that were photographed on both sides, and they were split into three groups based on outing type (see Methods): Aged (Table 1; *n* = 23), Oct22R (Table 2; *n* = 28), and Other (Table 3; *n* = 47). Count data on the number of spots on buffalofishes was best described by a negative binomial distribution. Combining all fully photographed buffalofishes that had either black or orange spots (Fig. 7), we found that the distribution of black spots was best explained by a negative binomial (mean = 9.31 ± 0.74, dispersion = 0.51 ± 0.08; ΔAIC_c_ = 0.0) compared to a Poisson (mean = 9.31 ± 0.31; ΔAIC_c_ > 350.0) (Fig. 7). Generalized regression with negative binomial error revealed the number of black spots did not differ across any of these three outing types (Aged, Oct22R, Other) pooling species (χ^2^ = 0.00, df = 2, *n* = 97, *p* = 0.9976, *R*^2^ = 0.00), nor by species: black buffalo (χ^2^ = 1.23, df = 2, *n* = 6 *p* = 0.5416, *R*^2^ = 0.17), bigmouth buffalo (χ^2^ = 1.12, df = 2, *n* = 68 *p* = 0.5722, *R*^2^ = 0.02) or smallmouth buffalo (χ^2^ = 0.11, df = 2, *n* = 23 *p* = 0.9486, *R*^2^ = 0.00). That is, the number of black spots on buffalofishes did not differ by fishing outing.

**Table 2:** Size and spot characteristics of buffalofishes from Apache Lake, Arizona caught and released during the October 2022 angling expedition (Oct22R) (*n* = 28).

Species	Capture Date	Mass		#B	#O	RC ID
*I. bubalus*	25-Oct-22	20.38		4	0	
*I. bubalus*	26-Oct-22	10.35		12	1	
*I. bubalus*	30-Oct-22	7.26		3	0	29
*I. cyprinellus*	27-Oct-22	12.30		10	0	
*I. cyprinellus*	27-Oct-22	10.15		5	1	26
*I. cyprinellus*	24-Oct-22	9.84		4	0	
*I. cyprinellus*	24-Oct-22	9.70		4	0	
*I. cyprinellus*	29-Oct-22	8.56		10	4	
*I. cyprinellus*	30-Oct-22	8.25		18	5	
*I. cyprinellus*	27-Oct-22	7.71		2	1	9
*I. cyprinellus*	28-Oct-22	7.65		4	6	30
*I. cyprinellus*	30-Oct-22	6.52		10	4	
*I. cyprinellus*	30-Oct-22	6.49		50	1	5
*I. cyprinellus*	28-Oct-22	6.41		8	2	
*I. cyprinellus*	29-Oct-22	5.73		16	2	
*I. cyprinellus*	29-Oct-22	5.64		5	1	
*I. cyprinellus*	29-Oct-22	5.39		3	4	3
*I. cyprinellus*	28-Oct-22	5.16		11	1	27
*I. cyprinellus*	27-Oct-22	5.05		5	0	
*I. cyprinellus*	28-Oct-22	4.76		7	7	
*I. cyprinellus*	29-Oct-22	4.71		18	44	
*I. cyprinellus*	27-Oct-22	4.45		5	1	
*I. cyprinellus*	28-Oct-22	4.14		21	15	
*I. cyprinellus*	27-Oct-22	3.91		3	0	
*I. cyprinellus*	29-Oct-22	3.88		6	0	
*I. cyprinellus*	24-Oct-22	NA		4	0	
*I. niger*	30-Oct-22	6.24		7	1	25
*I. niger*	30-Oct-22	5.90		4	1	

Data are sorted by species and then by size (mass in kg); #B = number of black spots; #O = number of orange spots; RC ID = Recapture ID (in conjunction with Table 4); NA = not available.

**Table 3:** Size and spot characteristics of the other caught and released buffalofishes from Apache Lake, Arizona that were photographed on both sides (*n* = 47).

Species	Capture date	Mass	#B	#O	RC ID
*I. bubalus*	20-Mar-23	13.66	5	0	
*I. bubalus*	13-Mar-22	12.16	3	0	
*I. bubalus*	22-Nov-22	11.79	6	0	
*I. bubalus*	22-Apr-23	11.62	3	0	
*I. bubalus*	1-Aug-19	11.25	3	0	
*I. bubalus*	12-Mar-22	10.46	10	0	
*I. bubalus*	21-Apr-23	10.26	12	3	
*I. bubalus*	13-Mar-22	8.22	7	0	28
*I. bubalus*	12-Mar-22	7.91	12	0	
*I. bubalus*	17-Jan-21	7.34	3	0	18
*I. bubalus*	12-Mar-22	7.34	1	0	
*I. bubalus*	14-Jul-18	7.17	12	0	1
*I. bubalus*	14-Mar-22	6.24	4	0	
*I. bubalus*	14-Mar-22	6.12	3	0	
*I. bubalus*	12-Mar-22	5.93	4	0	
*I. bubalus*	24-Oct-20	NA	2	0	
*I. bubalus*	10-Nov-22	NA	9	6	
*I. bubalus*	12-Nov-22	NA	3	1	
*I. cyprinellus*	12-Mar-22	13.01	10	0	
*I. cyprinellus*	24-Oct-20	9.98	6	1	15
*I. cyprinellus*	24-Oct-20	9.98	2	0	16
*I. cyprinellus*	21-Apr-23	9.3	26	0	
*I. cyprinellus*	24-Feb-19	9.02	12	1	4
*I. cyprinellus*	25-Apr-19	8.99	25	1	7
*I. cyprinellus*	13-Mar-22	8.87	47	3	
*I. cyprinellus*	22-Nov-20	7.97	40	9	17
*I. cyprinellus*	19-Feb-22	7.94	5	0	
*I. cyprinellus*	22-Apr-23	7.34	7	1	
*I. cyprinellus*	11-Dec-21	6.92	10	0	23
*I. cyprinellus*	21-Apr-23	6.44	5	1	
*I. cyprinellus*	25-Apr-19	5.9	4	5	
*I. cyprinellus*	14-Mar-22	5.78	2	0	
*I. cyprinellus*	24-Oct-20	5.61	4	1	11
*I. cyprinellus*	22-Apr-23	5.61	8	1	
*I. cyprinellus*	2-Aug-20	5.5	5	3	
*I. cyprinellus*	23-Apr-23	5.39	4	0	
*I. cyprinellus*	8-Nov-19	5.22	3	0	
*I. cyprinellus*	14-Mar-22	5.16	3	0	
*I. cyprinellus*	15-Aug-20	5.02	25	3	10
*I. cyprinellus*	19-Feb-22	4.99	30	8	31
*I. cyprinellus*	23-Apr-23	4.45	6	0	
*I. cyprinellus*	12-Mar-22	4.03	5	0	24
*I. cyprinellus*	11-Oct-21	2.38	2	0	22
*I. cyprinellus*	11-Nov-22	NA	7	0	
*I. cyprinellus*	21-May-23	NA	19	1	
*I. niger*	20-Mar-23	17.86	8	0	
*I. niger*	21-May-23	NA	8	0	

Data are sorted by species and then by size (mass in kg); #B = number of black spots; #O = number of orange spots; RC ID = Recapture ID (in conjunction with Table 4); NA = not available.

For orange spots we also found that the distribution was best explained by a negative binomial (mean = 2.09 ± 0.38, dispersion = 2.65 ± 0.54; ΔAIC_c_ = 0.0) compared to a Poisson (mean = 2.09 ± 0.15; ΔAIC_c_ > 250.0) (Fig. 7). The number of orange spots on buffalofishes did differ across the three outing types pooling species (χ^2^ = 10.47, df = 2, *n* = 97, *p* = 0.0053, *R*^2^ = 0.10), but this was due to an outlier individual. Namely, buffalofishes released at the Oct22R expedition had more orange spots than those assessed from the other outing types (parameter estimate = 1.25 ± 0.40, χ^2^ = 9.82, *p* = 0.0016), and Aged versus Other were not significantly different (parameter estimate = 0.82 ± 0.43, χ^2^ = 3.57, *p* = 0.0590). Conducting the analysis by species, the number of orange spots by outing type did not differ for black buffalo (χ^2^ = 1.29, df = 2, *n* = 6, *p* = 0.5241, *R*^2^ = 0.54) or smallmouth buffalo (χ^2^ = 0.08, df = 2, *n* = 23, *p* = 0.9606, *R*^2^ = 0.00). The exception was bigmouth buffalo orange spots (χ^2^ = 7.04, df = 2, *n* = 68, *p* = 0.0296, *R*^2^ = 0.09), where individuals caught and released at the October 2022 expedition (Oct22R) had significantly more orange spots (parameter estimate = 1.09 ± 0.41, χ^2^ = 6.96, *p* = 0.0083) than bigmouth buffalo in the other two groups (Aged versus Other were not significantly different: parameter estimate = 0.67 ± 0.45, χ^2^ = 2.26, *p* = 0.1331). A bigmouth buffalo caught at the October 2022 expedition had 44 orange spots, which was an extreme outlier (Fig. 7). Without this outlier, the Oct22R group effect for bigmouth buffalo orange spots was not significant (χ^2^ = 3.33, df = 2, *n* = 67, *p* = 0.1889).

Bigmouth buffalo are the most spotted of the three old-age (apparent or confirmed) buffalofish species at Apache Lake, and the number of the two types of spot (orange or black) on a given individual are weakly positively correlated with one another. Generalized regression assuming negative binomial error with species explained significant variation in the black spot distribution (χ^2^ = 9.97, df = 2, *p* = 0.0068, *R*^2^ = 0.09), and although black buffalo to smallmouth buffalo did not significantly differ in number of black spots (parameter estimate = 0.26 ± 0.35, χ^2^ = 0.53, *p* = 0.4634), bigmouth buffalo had more black spots than smallmouth buffalo or black buffalo (parameter estimate = 0.58 ± 0.19, χ^2^ = 9.63, *p* = 0.0019). In addition, generalized regression assuming negative binomial error with species explained significant variation in the orange spot distribution (χ^2^ = 17.25, df = 2, *p* = 0.0002, *R*^2^ = 0.14). Again, black buffalo to smallmouth buffalo did not significantly differ in orange spots (parameter estimate = − 0.45 ± 1.01, χ^2^ = 0.20, *p* = 0.6570), but bigmouth buffalo had more orange spots than smallmouth buffalo or black buffalo (parameter estimate = 1.67 ± 0.46, χ^2^ = 13.40, *p* = 0.0003). This result was robust with or without the outlier bigmouth buffalo that had 44 orange spots. We also found the number of black spots is weakly positively correlated with the number of orange spots for a bigmouth buffalo from Apache Lake (*r* = 0.25, *F*_1,66_ = 4.26, *p* = 0.0430). However, the number of each type of spot on a given individual was not correlated for smallmouth buffalo (*r* = 0.36, *F*_1,21_ = 3.13, *p* = 0.0912) or black buffalo (*r* = -0.37, *F*_1,4_ = 0.62, *p* = 0.4760).

The presence or absence (Y/N) of orange spots on apparently old-age or confirmed old-age Apache buffalofishes also significantly differs by species. A likelihood ratio test indicated that the distribution of orange spots between species was not random (χ^2^ = 14.19, df = 2, *n* = 97, *p* = 0.0008, *R*^*2*^ = 0.11). That is, the majority of bigmouth buffalo (65%) had orange spots whereas only 22% of smallmouth buffalo and 33% of black buffalo had orange spots. The presence or absence of black spots (Y/N) did not differ by species, as all apparently old-age or confirmed old-age individuals had black spots.

External orange and black spots are slowly accruing, unique long-lasting marks that allow for identification of recaptured buffalofishes across years. Across our database of known buffalofish capture events (cases) at Apache Lake (*n* = 222), 71 cases are derived from 31 recaptured individuals (26 bigmouth buffalo, 4 smallmouth buffalo, 1 black buffalo; Fig. 1, Table 4). Overall, there have been 27 buffalofishes caught exactly twice, 1 individual captured exactly three times, 2 captured exactly four times, and 1 individual caught six times (Table 4, Fig. 4). Therefore, according to our buffalofish photo database, a maximum total of 182 unique individuals have been caught by rod-and-line anglers at Apache Lake (128 bigmouth buffalo, 41 smallmouth buffalo, and 13 black buffalo) from 2018 to 2023. This is likely an overestimate of unique individuals because not all captured specimens were photographed on both sides by the angler. Interestingly, the recaptured buffalofishes were caught across time intervals as short as 12 days, to as long as 4.33 years and distances of up to 13 km separating capture locations (Table 4; Figs. 1, 4, 5, 6). There were 9 individuals captured across time intervals > 2 years (2.29–4.33 years), yet none displayed perceptible differences in their black or orange spots (size, number, or intensity of color) across these durations, even for relatively small spots (< 0.05 cm^2^) across intervals > 4 years (e.g., Fig. 6c-d).

**Table 4:** Buffalofish capture-recapture sequences from Apache Lake, Arizona, in chronological order of original capture (Case “a” for each recapture ID).

RC ID	Case	Date	Years FOC	Loc	Km FPC	Species	Sex	Mass (kg)	ΔMass FPC	Fish Age
1	a	14-Jul-18		CB		*I. bubalus*		7.17		
1	b	12-Nov-22	4.33	MAR	13.0			9.41	2.24	
2	a	17-Nov-18		LBC		*I. cyprinellus*	F	7.03		93
2	b	18-Nov-21	3.01	MAR	7.5			9.95	2.92	96
3	a	15-Jan-19		MAR		*I. cyprinellus*				
3	b	18-Mar-21	2.17	MAR	< 0.5			5.39		
3	c	11-Oct-21	2.74	MAR	< 0.5					
3	d	29-Oct-22	3.79	MAR	< 0.5			5.39	0.00	
4	a	24-Feb-19		MAR		*I. cyprinellus*		9.02		
4	b	11-Mar-22	3.04	MAR	< 0.5			10.21	1.19	
5	a	13-Apr-19		MAR		*I. cyprinellus*		5.56		
5	b	18-Mar-21	1.93	MAR	< 0.5			6.72	1.16	
5	c	11-Oct-21	2.50	MAR	< 0.5					
5	d	30-Oct-22	3.55	MAR	< 0.5			6.49	-0.23	
5	e	11-Nov-22	3.58	MAR	< 0.5					
5	f	23-Apr-23	4.03	MAR	< 0.5			6.83	0.34	
6	a	25-Apr-19		MAR		*I. cyprinellus*		8.99		
6	b	8-Nov-19	0.54	MAR	< 0.5			10.04	1.05	
7	a	25-Apr-19		MAR		*I. cyprinellus*		8.99		
7	b	23-Apr-23	4.00	MAR	< 0.5			10.21	1.22	
8	a	25-Apr-19		MAR		*I. cyprinellus*		3.43		
8	b	1-Aug-19	0.27	MAR	< 0.5					
8	c	7-Aug-21	2.29	MAR	< 0.5					
9	a	1-Aug-19		MAR		*I. cyprinellus*		5.30		
9	b	27-Oct-22	3.24	MAR	< 0.5			7.71	2.41	
10	a	15-Aug-20		MAR		*I. cyprinellus*				
10	b	13-Mar-22	1.58	MAR	< 0.5			5.02		
11	a	24-Oct-20		MAR		*I. cyprinellus*		5.61		
11	b	2-Oct-21	0.94	CW	0.5					
12	a	24-Oct-20		MAR		*I. cyprinellus*	F	8.62		85
12	b	19-Nov-21	1.07	MAR	< 0.5			9.16	0.54	86
13	a	24-Oct-20		MAR		*I. cyprinellus*	M	5.30		103
13	b	28-Apr-21	0.51	MAR	< 0.5			5.30	0.00	104
13	c	7-Aug-21	0.79	MAR	< 0.5					104
13	d	18-Nov-21	1.07	MAR	< 0.5			4.96	-0.34	104
14	a	24-Oct-20		MAR		*I. cyprinellus*	F	11.00		97
14	b	18-Nov-21	1.07	MAR	< 0.5			12.67	1.67	98
15	a	24-Oct-20		MAR		*I. cyprinellus*				
15	b	1-Apr-23	2.44	MAR	< 0.5			9.98		
16	a	24-Oct-20		LBC		*I. cyprinellus*				
16	b	28-Jan-22	1.26	LBC	< 0.5			9.98		
17	a	22-Nov-20		LBC		*I. cyprinellus*				
17	b	21-Nov-21	1.00	LBC	< 0.5			7.97		
18	a	17-Jan-21		MAR		*I. bubalus*		7.34		
18	b	10-Mar-22	1.14	MAR	< 0.5			7.71	0.37	
19	a	13-Feb-21		MAR		*I. cyprinellus*	M	5.95		101
19	b	20-Nov-21	0.77	MAR	< 0.5			6.10	0.14	101
20	a	18-Mar-21		MAR		*I. cyprinellus*	M			98
20	b	20-Nov-21	0.68	MAR	< 0.5			3.91		98
21	a	7-Aug-21		MAR		*I. cyprinellus*				
21	b	27-Nov-21	0.31	LBC	7.5			6.61		
22	a	11-Oct-21		MAR		*I. cyprinellus*		2.38		
22	b	14-Mar-22	0.42	MAR	< 0.5			2.32	-0.06	
23	a	11-Dec-21		MAR		*I. cyprinellus*		6.92		
23	b	22-Apr-23	1.36	MAR	< 0.5			6.92	0.00	
24	a	12-Mar-22		MAR		*I. cyprinellus*		4.03		
24	b	22-Apr-23	1.11	MAR	< 0.5			4.45	0.43	
25	a	12-Mar-22		MAR		*I. niger*		6.46		
25	b	30-Oct-22	0.64	MAR	< 0.5			6.24	-0.23	
26	a	13-Mar-22		MAR		*I. cyprinellus*		10.69		
26	b	27-Oct-22	0.62	MAR	< 0.5			10.15	-0.54	
27	a	13-Mar-22		MAR		*I. cyprinellus*		5.33		
27	b	28-Oct-22	0.63	MAR	< 0.5			5.16	-0.17	
28	a	13-Mar-22		MAR		*I. bubalus*		8.22		
28	b	21-Apr-23	1.11	MAR	< 0.5			8.08	-0.14	
29	a	14-Mar-22		MAR		*I. bubalus*		10.26		
29	b	30-Oct-22	0.63	MAR	< 0.5			7.26	-3.01	
30	a	28-Oct-22		MAR		*I. cyprinellus*		7.65		
30	b	21-Apr-23	0.48	MAR	< 0.5			6.69	-0.96	
31	a	19-Feb-22		MAR		*I. cyprinellus*				
31	b	11-Mar-22	0.05	MAR	< 0.5			4.99		

RCID = recapture ID; FOC = from original capture; Loc = location: CB = Chunk Beach, MAR = Marina, Crabtree Wash, LBC = Lower Burnt Corral; UBC = Upper Burnt Corral; Km FPC = kilometers from previous capture; Sex = Female (F) and Male (M); ΔMass FPC = change in mass (in kg) from previous capture; Age in years estimated from otoliths. NA = not available.

Gonadal indices of buffalofishes at Apache Lake were generally indicative of robust gonadal investment. For the buffalofishes that were dissected for age analysis, GSIs for 8 of 13 males ranged from 5.4 to 9.3% (other males ranged from 1.0 to 3.6% GSI). For the remaining 10 females that were dissected for age analysis, 9 had ovaries that were well-developed with GSI values ranging from 14.1 to 22.3%. The outlier female had a GSI of 3.3% and ovaries were composed primarily of fatty tissue.

## Discussion

The longevity of *Ictiobus* is exceptionally rare. With a sample size of only 23 individuals across the three species of buffalofishes at Apache Lake, we found direct evidence of centenarian longevity for black buffalo (108 years), bigmouth buffalo (105 years), and smallmouth buffalo (101 years). Prior to this study, there were approximately 35 animal species worldwide with documented lifespans of more than 100 years, and only one genus of animal (*Sebastes*: the ocean rockfishes) with three or more species known to live beyond a century^68,69^. Thus, the longevity of *Ictiobus* can be considered extraordinary. Fishes provide an excellent opportunity to understand age demographics of wild populations because most species can be aged directly via the thin-sectioned otolith^47,56^. Indeed, this technique also allows for age validation of long-lived species^47^, which has been rigorously demonstrated for buffalofishes^16,19,33^.

The natural age ceiling for all buffalofish species is likely substantially older than what is currently known. Prior to 2019, no buffalofish species was known to live more than 26 years^16^. In the past four years buffalofishes have gained substantial study and attention, and as a result, are amidst a paradigm shift in the way we humans understand them. The bigmouth buffalo has been discovered to exhibit a slow pace of life, pronounced episodic recruitment^16,19,40^, negligible senescence, improvements in physiological systems at 100 years old^44^, and a maximum reported longevity of 127 years from the first sample (*n* = 52 individuals) analyzed from Canada^40^. In Arizona, we found ages that nearly double the previously reported maximum age of 56 years for black buffalo^16^, and increase the maximum longevity known for smallmouth buffalo (62 years)^45^ by several decades. The maximum ages for smallmouth buffalo and black buffalo that we document in this study are more than 80 years older than the maximum reported ages for these species prior to 2019^1,46^. The fleshylip buffalo *Ictiobus labiosus*, native to Mexico, and the usumacinta buffalo *Ictiobus meridionalis*, which is native Mexico and Guatemala, are both large-bodied freshwater fishes for which there is no published age information. We recommend these two species are investigated for otolith-derived age demographics. The genus *Ictiobus* likely contains valuable information on how vertebrate lineages may evolve to postpone senescence^44,70^.

Evidence indicates that individual buffalofishes from the Arizona stocking in 1918 are likely still alive as of 2023. Of the 23 otolith-aged fish, there were 4 individuals across two species (bigmouth buffalo and black buffalo) that had estimated year classes ranging from 1914 to 1917. These year classes are consistent with possible hatch years of the ~ 420 buffalofish fingerlings, yearlings, and adults that were transported by rail from rearing ponds along the Mississippi River to Roosevelt Lake in 1918^11^, and considered to have originated from the Fairport Biological Station in Iowa^26^. This Station contained a rearing lab and more than a dozen rearing ponds alongside the Mississippi River, and the workforce at the Fairport Biological Station pioneered buffalofish rearing and were well known for their success^10,12,14^. Buffalofishes were highly esteemed food fish in the late 19th and early twentieth century, and their abundance had drastically diminished because of overharvest by commercial fishing^12,13,15^. This caused widespread concern such that the Fairport Biological Station, which was funded by an Act of Congress in 1908 and was not formally opened until August of 1914^12^, began rearing buffalofishes in the spring of 1915^9^. Members of the Station’s force and commercial harvesters eagerly worked together to extract gametes from spawn-ready adult buffalofishes each spring from 1915 to 1917, and eggs and milt were collected from the three different species that exist sympatrically in the Mississippi River basin^9,13,15^. Fingerlings, yearlings, and 2-year-old buffalofishes became intermixed in the ponds by 1917^9,10,15^. In addition, adult buffalofishes were collected from the nearby Mississippi River in each of these years and added to the ponds in an effort to induce natural reproduction in the ponds themselves^9,13,15^. Adult buffalofishes did not reproduce in the ponds in 1915 and 1916 under stagnant water-level conditions, but in 1917 workers artificially increased water levels along with rising temperatures in the spring, and finally the buffalofishes spawned^9,13,15^. In December of 1917 the Station building (where the artificial propagation of the eggs took place) burned down, which was not rebuilt until 1920^15^. Despite this loss, the workforce continued to maintain the rearing ponds^15^. Therefore, a mix of 1–3 year-old buffalofishes existed in the rearing ponds in 1918 plus the possibility of adults of varying ages^15^. The estimated 420 fingerlings, yearlings, and adults that were sent to Globe, Arizona in 1918^11^ were likely composed of these individuals.

Presuming angler catch approximates the buffalofish populations at large, the age-structure of bigmouth buffalo from Apache Lake reveals an extremely long-lived population that is, in general, a century old. Although we document fish as young as 85–86 years of age in 2021 (the 1935 and 1936 year classes), evidence suggests most (~ 90%) bigmouth buffalo in Apache Lake hatched during the early 1920s or earlier and that recruitment has been episodic. Indeed, evidence suggests that most bigmouth buffalo in Apache Lake are 100 years or older as of 2023. In addition, most of the smallmouth buffalo and black buffalo directly aged from Apache Lake hatched during the 1930s or earlier. Interestingly, small buffalofishes in Apache Lake during the 1960s were conspicuously absent despite thorough sampling across a variety of nets and gears, which led researchers to speculate that there was a lack of recruitment^25,29^. This is consistent with the 74-year recruitment gap (no recruits from year classes 1940s–2000s) we observe for buffalofishes in Apache Lake. In addition, the relative abundance of bigmouth buffalo: smallmouth buffalo: black buffalo was approximately 4:2:1 in Apache Lake during this time^26^, which is consistent with the rank order of abundance that we document from the angler catch. Collectively this supports the notion that the angler catch of buffalofishes from Apache Lake approximates the community at large. Apache Lake was not formed until 1927 after the completion of Horse Mesa Dam^23^. Thus, for any of the buffalofishes currently found in Apache Lake, it is not known when they moved downstream of Roosevelt Dam, or if some of the progeny were actually spawned downstream of Roosevelt Dam (pre-1927) or in Apache Lake (post-1927). However, it is known that buffalofishes were present in the Salt River chain of lakes by the 1930s^28^.

One potential hypothesis for the dominant early-1920s cohort of bigmouth buffalo found in Apache Lake is that they hatched during trophic upsurge^71^ of the reservoirs. Trophic upsurges may have occurred in the Salt River chain of lakes as the dams were built and water levels rose to capacity for the first time during the 1910s–1920s^23^. Trophic upsurge has been documented to cause booms in recruitment for other long-lived freshwater fish^72^. Furthermore, water-level management practices in recent decades (for increased water-level stability)^23^ may be minimizing buffalofishes’ ability to successfully spawn or recruit in Apache Lake, as it is known that fluctuating water levels in spring are important for buffalofish spawning and recruitment^19,40^. Nonetheless, the trophic upsurge hypothesis is complicated by the fact that even if buffalofish spawn, variables such as the post-peak water-level recession rate may have undue influence on whether successful recruitment occurs^40^. Hypotheses of early recruitment in this system are also complicated because it is unknown what proportion of fingerlings: yearlings: adults were stocked in 1918, as well as when sexual maturity would have occurred for transplanted juvenile buffalofishes into Arizona. Clearly, otolith-derived population demographics of buffalofishes found in Roosevelt Lake and throughout the Salt River chain of lakes is needed.

Buffalofishes dissected for age analysis generally exhibited gonadal tissue indicative of individuals prepared to spawn. GSIs for 8 of 13 males were > 5%, which is typical of spawn-ready males^16,19^. In addition, several males expressed milt with slight pressure applied near the vent and some partial tuberculation was also evident, which is common for buffalofishes during the fall across their range. Likewise, 9 of 10 females had robust ovaries with GSIs > 14%. Overall, this suggests both males and females within the buffalofish populations of Apache Lake are capable of spawning. However, buffalofish spawning has not been confirmed in Apache Lake and should be investigated by species.

Analysis of black and orange spots on Apache Lake buffalofishes reveals broader insight into the age demographics of the buffalofish community. Only 1% of buffalofishes in Apache Lake lack black and orange spots according to a sample size of 98 individuals photographed on both sides. This single specimen (a smallmouth buffalo) was captured during the 2021 angling expedition and was 11 years old. All other buffalofishes directly aged (*n* = 22) had black or orange spots and ranged from 85 to 108 years old, and all other buffalofishes photographed on both sides (*n* = 75) had black or orange spots as well. We found no significant difference in the number of spots across all individuals that were fully photographed (except for one individual with more than 40 spots discussed below), which suggests the age distribution for the 75 released fish is consistent with the buffalofishes that had spots and were directly aged (i.e., likely > 80 years). There was one very large smallmouth buffalo (20.38 kg) in the dataset. Interestingly, it is known that bigmouth buffalo exhibit incredible variation in adult size at a given age within a given sex^16,19,40^. This 20.38 kg smallmouth buffalo is likely an exceptionally large female within the common old-age cohort of buffalofishes in Apache Lake.

Lackmann et al.^16^ found that black and orange spots both start appearing on bigmouth buffalo at ~ 40 years of age, that they were more accentuated or numerous in the oldest individuals (> 80 years), and that black spots (but not always orange spots) were always present on older-age (> 45 years) individuals. For populations of bigmouth buffalo in North Dakota^19^ and Canada^40^ this overall age-spot pattern has also been observed. Evidence from Apache Lake is consistent with these data and observations, and it appears to be the case for all buffalofish species. Indeed, not only were black or orange spots present, but spots were generally large, numerous, or intense in color. Overall, this suggests that young buffalofishes (< 40 years) in Apache Lake are rare and that > 90% of the buffalofish community in Apache Lake may be at least 40 years old, and possibly older. These findings reveal the potential for black or orange spots to serve as important and non-lethal indicators for approximate age in these fishes across their range. In addition, black or orange spots may be ubiquitous across the Ictiobinae as black spots were recently found to correlate with age in *Carpiodes*^34^, the only other extant genus in this subfamily.

Bigmouth buffalo are the most spotted of the Apache Lake buffalofishes and this is possibly due to their unique ecology. We found that black spots and orange spots are more numerous on bigmouth buffalo compared to smallmouth buffalo or black buffalo, and that even the categorical presence (Y/N) of orange spots on bigmouth buffalo was more frequent compared to the other two species. We hypothesize that this is due to fundamental differences in the ecology of bigmouth buffalo compared to the other two species. Bigmouth buffalo are known as pelagic filter feeders^24,57^ that bask in the near-surface sun in open water on relatively calm days^57^. Consuming a diet rich in cladocerans and various forms of phytoplankton including diatoms^24,57^, Apache Lake bigmouth buffalo likely consume proportionately more carotenoids^73^ than smallmouth buffalo or black buffalo that feed primarily on benthic invertebrates^24^. Moreover, there is individual variation in feeding behavior^24^. There was one bigmouth buffalo released at the October 2022 angling expedition (Oct22R) that had 44 orange spots, which was dozens more compared to any individual within the other groups of fully photographed individuals (Aged and Other). Furthermore, black spots were also more numerous on bigmouth buffalo compared to the other two species of buffalofishes. Spending more time feeding or basking in the open water sun, and less time near the bottom^24,57^, bigmouth buffalo are predisposed to a carotenoid-rich diet while also spending time under intense sun exposure. Lackmann et al.^16^ hypothesized that orange spots slowly accrue on the epidermis of bigmouth buffalo because of diet, and that black spots accrue across decades of sun exposure (melanosis). Evidence from Apache Lake indirectly supports these hypotheses. It is unknown what biological function (if any) these orange or black spots have, though we speculate orange spots may have evolved to give honest indication (to conspecifics) of vitality in advanced age. The underlying mechanisms of the formation of buffalofish orange and black spots should be further investigated.

Black or orange markings on old age buffalofishes allow for enhanced identification of individuals across multiple recapture events and years. Much like long-lived whales that can be identified by unique patterns on their flukes^59,74^, buffalofish black and orange spots enhance the ability to identify individuals and can be used as way to track individual movements, size fluctuations, age, recaptures, and perhaps eventually, population size. However, since both black and orange spots generally do not begin appearing until the approximate age of 40 years^16^, this enhanced ability to identify individual buffalofishes is likely not applicable to younger age buffalofishes < 40 years. For example, in some systems, younger age (< 40 years) buffalofishes are common as of the 2020s^17,19^. However, like what has been found in Apache Lake, there are other contemporary populations discovered where buffalofish > 40 years old compose the vast majority of a given population^16,40^. Furthermore, it is unknown if relatively young populations today could become proportionately old in a few decades, especially since buffalofishes are known to exhibit highly irregular recruitment^16,17,19,40^. Thus, the ability to identify buffalofish individuals through natural age spots likely has broad utility if high quality photographs are taken. Indeed, with dozens of recaptures and some Apache Lake individuals captured more than 4 years apart, up to six times, and still with no perceptible changes in spot pigmentation, it is abundantly clear that individuals can be identified via their natural markings and that black and orange spots accrue very slowly across buffalofishes’ long lifespans. Evidence indicates that if recapture events are interspersed at least once every several years, it may be possible to track buffalofish individuals for decades using their unique age markings and features. This could be tested in Apache Lake.

Our study demonstrates the value of collaborating with citizen scientists and recreational anglers, even when there is no funding. For more than a century, buffalofishes have survived in Arizona. Little did we know that some individuals have been alive all this time, persisting in the desert sanctuary of Apache Lake. As evidenced by the enthusiastic conservation angling community in Arizona, the exceptional lifespans of the buffalofishes unparalleled in freshwater fishes, and the ability to consistently recapture uniquely marked centenarians, it is clear buffalofishes offer great potential for anglers and researchers to uncover profound insights into freshwater biological systems. Thus, it is recommended that substantial effort is made to proactively study, manage, and protect these remarkable century-old fishes across North America.

### Supplementary Information

Supplementary Figure 1.

## Data Availability

The datasets generated and analyzed during the current study are available from the corresponding author on reasonable request.
