# Supplementary material for: Centenarian lifespans of three freshwater fish species in Arizona reveal the exceptional longevity of the buffalofishes (Ictiobus)
Source: Sci Rep. 2023 Oct 20;13:17401. doi: 10.1038/s41598-023-44328-8 (PMC10589290; doi:10.1038/s41598-023-44328-8)
Supplement: Supplementary file 1 — Supplementary Figure 1. [file 41598_2023_44328_MOESM1_ESM.docx]

**Centenarian lifespans of three freshwater fish species in Arizona reveal the exceptional longevity of the buffalofishes (*Ictiobus*)**

Alec R. Lackmann^1,3^*, Stuart A. Black^2^, Ewelina S. Bielak-Lackmann^3^, Jeffrey A. Lackmann^4^

^1^University of Minnesota Duluth, Department of Mathematics and Statistics, 140 Solon Campus Center, 1117 University Drive, Duluth, MN, 55812, USA; [alackman@d.umn.edu](mailto:alackman@d.umn.edu)

^2^Conservation angler, Phoenix, Arizona, USA; stuart@buffalogill.com

^3^ University of Minnesota Duluth, Department of Biology, 1035 Kirby Drive, SSB 207, Duluth, MN, 55812, USA; [alackman@d.umn.edu](mailto:alackman@d.umn.edu); ewelinabielak90@gmail.com

^4^North Dakota State University, Department of Biological Sciences, Dept. 2715, PO Box 6050, Fargo, ND, 58108, USA; jeffrey.lackmann@ndsu.edu

*corresponding author: [alackman@d.umn.edu](mailto:alackman@d.umn.edu)

Supplementary Information


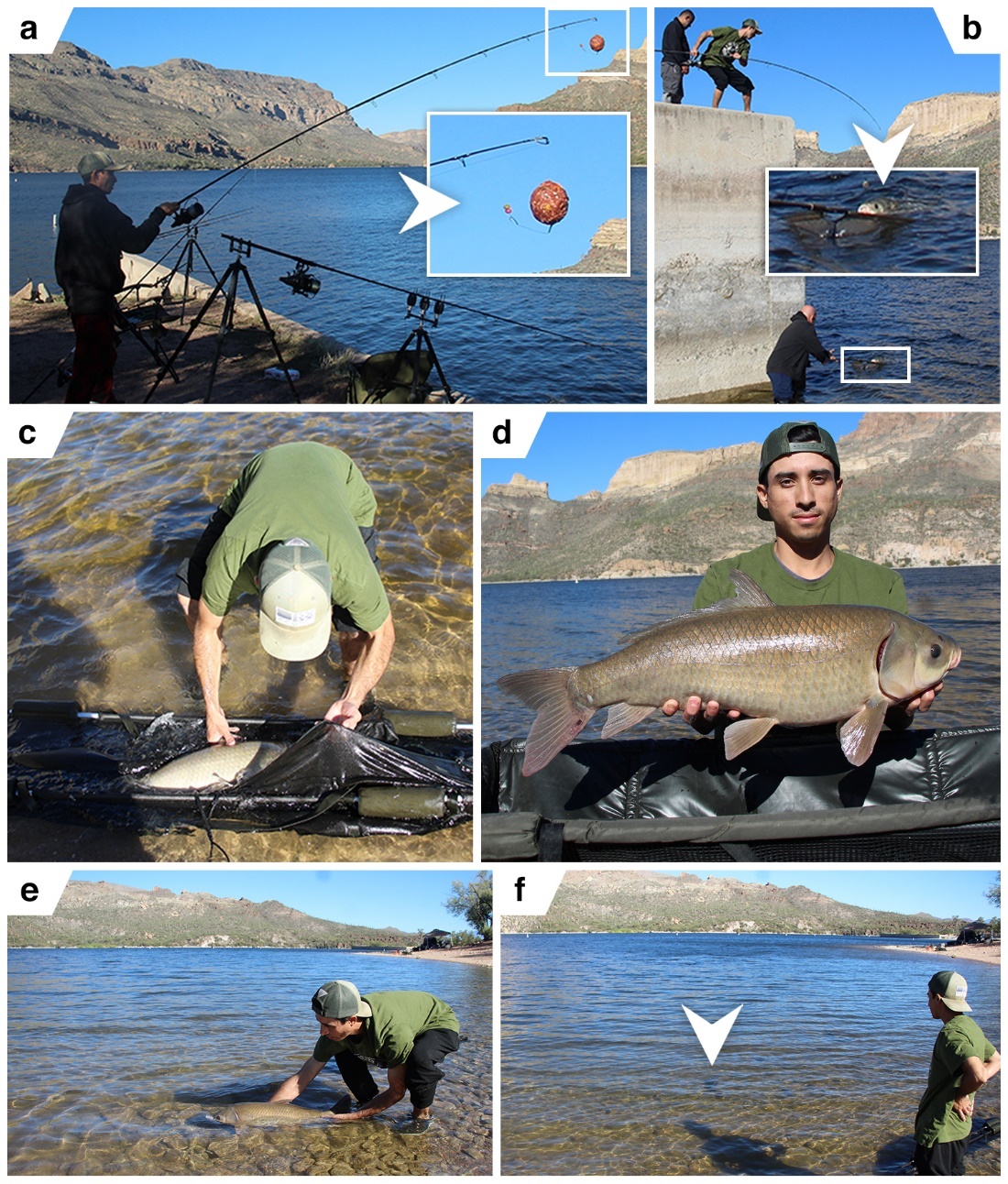


**Supplementary Figure 1.** Catch-photo-release buffalofish angling at Apache Lake, Arizona. **a** An angler prepares to cast his line consisting of pack-bait (sphere-like ball of ground bait packed around a weight), and a hair-rigged hooklink featuring sinking braid, a single hook and tethered fake corn kernels for bait (inset and arrow). **b** The fish (arrow) is landed with a large soft mesh landing net (inset) and **c** transferred to a retention sling until the cradle is ready. **d** The angler holds a bigmouth buffalo *Ictiobus cyprinellus* for pictures safely above the fish cradle. **e** The angler gently releases the fish and **f** looks on as it swims away (arrow).
